# Association between serum interleukin-6 concentrations and chronic obstructive pulmonary disease: a systematic review and meta-analysis

**DOI:** 10.7717/peerj.1199

**Published:** 2015-08-27

**Authors:** Jia Wei, Xiao-feng Xiong, Yi-hua Lin, Bi-xia Zheng, De-yun Cheng

**Affiliations:** 1 Department of Respiratory Medicine, West China Hospital of Sichuan University, Chengdu, Sichuan, China; 2 Department of Respiratory Medicine, First Affiliated Hospital of Xiamen University, Xiamen, Fujian, China; 3 Department of Respiratory Medicine, Third People’s Hospital, Chengdu, Sichuan, China

**Keywords:** Meta-analysis, Chronic obstructive pulmonary disease, Systemic inflammation, Interleukin-6, Pulmonary function

## Abstract

**Background.** Interleukin-6 (IL-6) is an important pro-inflammatory cytokine and has been implicated to play a role in the systemic inflammation of patients with chronic obstructive pulmonary disease (COPD). We conducted this meta-analysis to assess the association between serum IL-6 concentrations and COPD.

**Methods.** PubMed and Embase were searched for eligible studies. Data were extracted by two investigators (Wei J, Xiong XF) independently and analyzed using Review Manager 5.3 and STATA 12.0 software. Standard mean differences (SMDs) and 95% confidence intervals (CI) were calculated.

**Results.** Thirty-three studies were included in this meta-analysis. The serum IL-6 concentrations were higher in patients with stable COPD than healthy controls (SMD = 0.65, 95% CI [0.51–0.79]). COPD patients without major comorbidities also showed higher IL-6 levels than healthy controls (SMD = 0.74, 95% CI [0.56–0.91]). COPD patients with an forced expiratory volume in one second (FEV_1_) of either <50% predicted or >50% predicted had increased IL-6 concentrations compared to healthy controls (SMD = 0.77, 95% CI [0.48–1.05], SMD = 1.01, 95% CI [0.43–1.59], respectively). The serum IL-6 concentrations between mild-moderate and severe-very severe COPD patient groups were not found to be significant (SMD = −0.1, 95% CI [−0.65–0.44]).

**Conclusions.** This meta-analysis indicated that patients with stable COPD had higher serum IL-6 concentrations than healthy controls. No evidence showing positive or negative association between IL-6 concentrations and the severity of pulmonary function impairment was found. The correlation between IL-6 levels and pulmonary function was weak in different severities of stable COPD patients.

## Introduction

Chronic obstructive pulmonary disease (COPD) is one of the major causes of morbidity and mortality throughout the world. It is characterized by persistent airflow limitation that is usually progressive and associated with an enhanced chronic inflammation in the respiratory system (Vestbo et al., 2013). Once considered a respiratory disease, COPD is now regarded as a systemic inflammatory disease, and systemic complications may contribute to the disease outcome. Systemic inflammation is recognized as a risk factor for various extra-pulmonary complications including cachexia (Kotler, 2000), atherosclerosis (Ross, 1999) and anorexia (Johnson et al., 2002). Since evidence has showed that a number of different inflammatory cells and mediators play roles in the progression of COPD, interleukin-6 (IL-6) has become a subject of considerable research in recent years (Garcia-Rio et al., 2010). IL-6 is mainly secreted by *T* cells and macrophages. It is an interleukin that acts both as a pro-inflammatory cytokine and an anti-inflammatory myokine. A recent longitudinal study on inflammatory markers in COPD patients over three years indicates that elevated IL-6 levels in serum is predictive of increasing mortality in COPD (Celli et al., 2012). Moreover, a number of studies have shown that patients with COPD had higher serum concentrations of IL-6 than healthy controls (Donaldson et al., 2009; Garcia-Rio et al., 2010; Johnson et al., 2002; Ross, 1999). The potential importance of IL-6 in the pathogenesis of COPD is suggested by studies showing that high levels of serum IL-6 are associated with impaired or a rapidly declining lung function (Donaldson et al., 2009; Walston et al., 2007). Similarly, another study supports the association between elevated IL-6 levels and poor clinical outcomes in COPD patients (Agusti et al., 2012). However, results from different studies are not consistent. Also, most of the studies undertaken to evaluate this potential relationship were small in size (Celli et al., 2012; Walston et al., 2007), and may lack sufficient statistical power to address this issue adequately. Therefore, whether or not serum IL-6 concentrations are higher in COPD patients than in controls remains controversial. In an effort to overcome these limitations and have a better understanding of the relationship between serum IL-6 concentrations and COPD, a systemic review and meta-analysis is necessary.

## Methods

### Identification of studies

Two investigators (Wei J, Xiong XF ) independently searched PubMed and Embase until March 2015 to identify potentially relevant articles. The following search terms were used both in key words and free text words: (‘Pulmonary Disease, Chronic Obstructive’ [MeSH Terms] or ‘chronic obstructive pulmonary disease’ or ‘COPD’ or ‘COAD’ or ‘chronic obstructive airway disease’ or ‘chronic obstructive lung disease’ or ‘Chronic Airflow Obstructions’ or ‘Chronic Airflow Obstruction’) and (‘Interleukin-6’ [MeSH Terms] or ‘interleukin 6’ or ‘IL 6’) and (‘humans’ [MeSH Terms]). IL-6 in the blood was retrieved manually. There was no restriction on languages and references of all selected articles were retrieved to identify other relevant studies.

### Inclusion and exclusion criteria

Inclusion criteria were as follows: (1) prospective or retrospective observational studies reporting the serum concentrations of IL-6; (2) COPD patients who met the criteria of the American Thoracic Society (ATS) or European Respiratory Society (ERS) or Global Initiative for Chronic Obstructive Lung Disease (GOLD); (3) healthy controls with no medical illness or abnormalities. Exclusion criteria were as follows: (1) patients who were in an acute phase or were hospitalized; (2) patients with a history or diagnosis of asthma, allergy or other respiratory diseases other than COPD; (3) articles with no original data.

### Data extraction

While the titles and abstracts met the inclusion criteria, full articles were searched and screened by two investigators (Wei J, Xiong XF) to confirm eligibility independently. Disagreements were resolved by consensus or a third opinion (Cheng DY). The following information was extracted: first author, year of publication, original country, sample size, age, smoking status, GOLD stages of cases, mean value, standard difference (SD) , standard error of mean (SEM) and 95% confidence intervals (CI) of both COPD patients and healthy subjects. The SEM or 95% CI was transformed into SD using statistical formulas.

### Statistical analysis

All the data were analyzed using Review Manager (version 5.3, The Nordic Cochrane Centre, The Cochrane Collaboration, Copenhagen, Denmark) and STATA 12.0 software (StataCorp LP, College Station, Texas, USA). Weighted mean differences (WMDs) were selected to combine statistics. If the difference in mean values was too large or resulted in large heterogeneity, the standard mean differences (SMDs) values were selected. The *I*^2^ statistic was used for quantifying heterogeneity. *I*^2^ < 25%, 25–75% and >75% was thought to indicate low, moderate and high heterogeneity (Higgins & Thompson, 2002). If the *P*-value for the heterogeneity was >0.05, which showed low heterogeneity (*I*^2^ < 25%), then the fixed-effects model was selected; otherwise, the random-effects model was applied (DerSimonian & Laird, 1986). A two-tailed *Z*-test was performed to statistically assess differences between healthy subjects and a disease stage, with *P* < 0.05 considered statistically significant. In order to assess some major comorbidities and the lung function effects, subgroup analyses were performed according to the comorbidities of participants and the GOLD stages of the cases (GOLD stage 1–2 subgroup and GOLD stage 3–4 subgroup). Sensitivity analysis was performed by removing one study each time to see whether the results of the rest would be affected. Egger’s test and funnel plot were used to assess the publication bias (Egger et al., 1997).

## Results

### Characteristics of the studies

**Figure 1:**
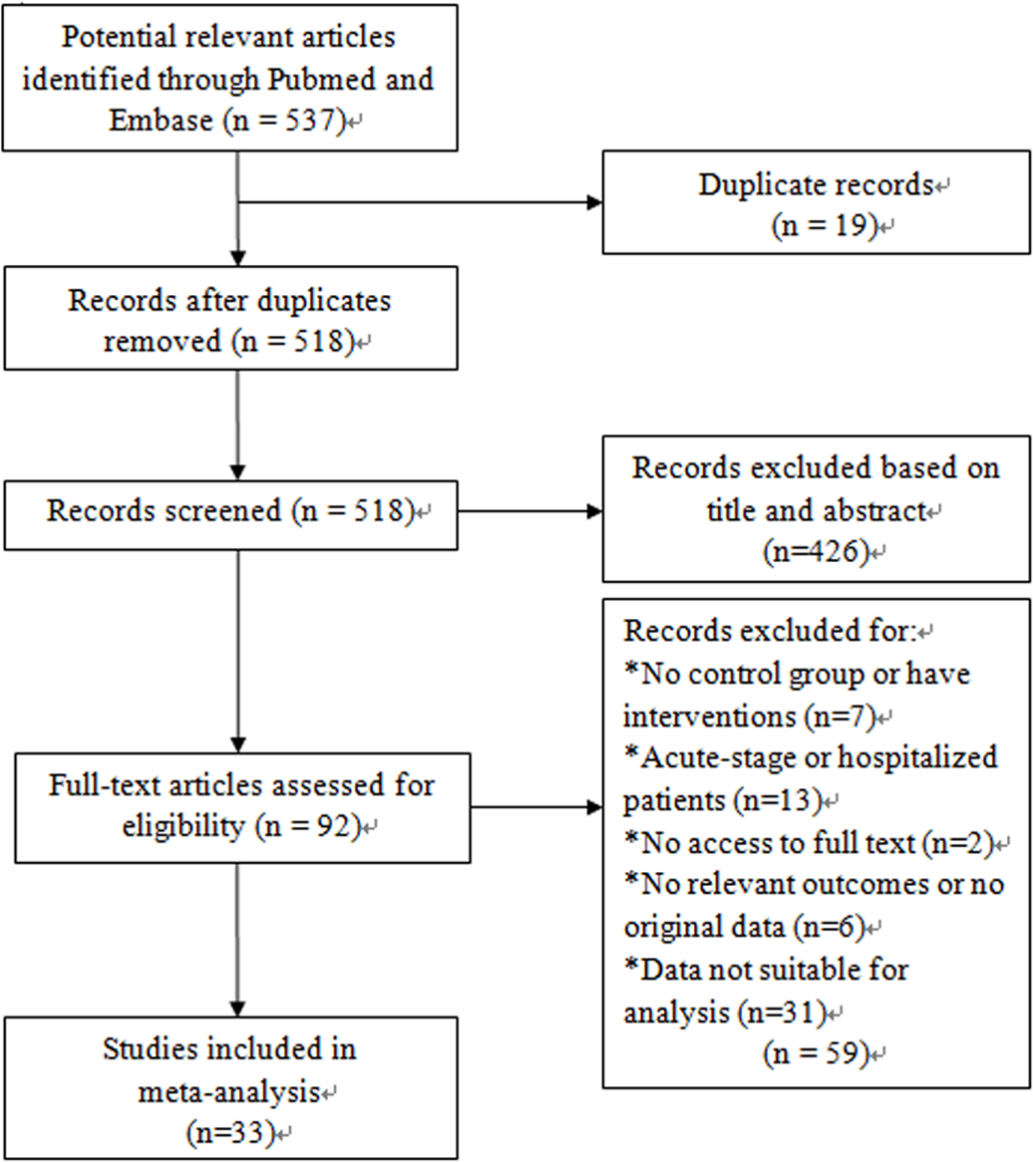
Flow diagram of study indentification, inclusion and exclusion.

We identified 537 relevant studies that fit our search strategy. The procedure for identifying and selecting eligible studies is shown in Fig. 1. Of all the potential studies, 19 duplicate records were removed, leaving 518 articles for screening. 426 articles were excluded by screening titles and abstracts. Two articles were not accessible and only 90 were available for full-text reading. After reading the full text, 26 articles were excluded according to the exclusion criteria. Among the 64 articles, another 30 were excluded as the data were presented as median (range) and were not suitable for meta-analysis. One study had a standard differential with zero, so the data was excluded. Finally, a total of 33 articles were included for our systematic review and meta-analysis (Aaron et al., 2010; Bai et al., 2011; Bolton et al., 2007a; Bolton et al., 2007b; de Moraes et al., 2014; Debigare et al., 2003; Foschino Barbaro et al., 2007; Gagnon et al., 2014; Gale et al., 2011; Hacker et al., 2009; Hageman et al., 2003; He et al., 2010; Higashimoto et al., 2008; Huertas et al., 2010; Itoh et al., 2004; Jammes et al., 2008; Ju & Chen, 2012; Karadag et al., 2008a; Karadag et al., 2008b; Koechlin et al., 2004; Oncel et al., 2010; Palange et al., 2006; Rabinovich et al., 2003; Sabit et al., 2007; Tomoda et al., 2007; Uzum et al., 2014; Van Helvoort et al., 2006; Vogiatzis et al., 2007; Walter et al., 2008; Wang et al., 2014; Yasuda et al., 1998; Yende et al., 2006; Ying et al., 2008). The basic characteristics of all the included studies are listed in Table 1.

**Table 1:** The characteristics of studies included in the meta-analysis.

Study (first author, year)	Country	Age(mean/range)		Numbers		Serum IL-6 levels (means ± SD)(pg/ml)		Smoking status of subjects		GOLD stages of cases
		Cases	Controls	Cases	Controls	Cases	Controls	Cases	Controls	
Yasuda 1998	Japan	66	66	39	22	15.23 ± 18.35	2.1 ± 1.41	Mixed	Mixed	GOLD I–III
Sabit 2007	UK	64.9	62.0	75	42	2.11 ± 1.84	1.29 ± 1.58	≥5 pack-year	Smokers	GOLD I–IV
Huertas 2010	Italy	68.54	63	39	12	2.75 ± 1.38	1.2 ± 0.69	Mixed	Nonsmokers	GOLD II–IV
Richard 2003	Canada	67.4	65	45	16	4.92 ± 3.22	2.1 ± 1.2	Mixed	Ex-smokers	No stage information
Bolton 2007a	Netherlands	Not mentioned		40	18	4.03 ± 1.9	1.71 ± 1.7	Mixed	Mixed	GOLD II–IV
Palange 2006	Italy	68	63	18	12	3.7 ± 1.7	2.5 ± 1.2	Ex-smokers	Nonsmokers	GOLD II
Wang 2014	China	73.1	74.5	58	29	39.21 ± 27.34	23.41 ± 12.86	Mixed	Mixed	GOLD I–IV
Moraes 2014	Brazil	66.43	63.7	50	16	28.2 ± 29.86	5.42 ± 3.72	≥10 pack-year	Nonsmokers	GOLD I–IV
Bai 2011	China	65.12	58.4	80	20	0.45 ± 0.12	0.35 ± 0.083	Smokers	Smokers	No stage information
Gale 2011	UK	49–80	51–81	32	20	3.8 ± 2.1	1.3 ± 3.1	≥5 pack-year	≥5 pack-year	GOLD II–IV
Koechlin 2004	France	60	58	10	7	9.46 ± 9.2	1.44 ± 0.53	Ex-smokers	Nonsmokers	GOLD II–III
Vogiatzis 2007	Greece	66	61	15	10	5.68 ± 3.76	0.76 ± 0.54	Not mentioned	Not mentioned	GOLD III–IV
Tomoda 2007	Japan	70.93	69.3	31	12	2.23 ± 1.27	1.6 ± 0.3	Not mentioned	Not mentioned	No stage information
Itoh 2004	Japan	71	69	50	13	3.34 ± 3.20	1.6 ± 1.08	Mixed	Mixed	GOLD I–IV
Jammes 2008	France	53	48	17	18	5.87 ± 2.06	3.31 ± 2.29	Not mentioned	Mixed	No stage information
Van 2006	Netherlands	65.5	59	20	10	2.8 ± 2.68	1.9 ± 1.9	Ex-smokers	Nonsmokers	GOLD I–IV
Rabinovich 2003	Spain	65	63	11	6	7.9 ± 8.0	6.1 ± 9.3	Not mentioned	Mixed	No stage information
Foschino 2007	UK	52	49	27	15	9.18 ± 2.31	5.82 ± 0.98	Ex-smokers	Ex-smokers	GOLD I
Yende 2006	USA	73.6	73.2	268	2005	2.6 ± 1.9	2.2 ± 1.8	Mixed	Mixed	GOLD I–IV
Bolton 2007b	Netherlands	66.7	62.9	56	29	2.75 ± 1.87	1.43 ± 2.12	Smokers	Mixed	No stage information
HE 2010	China	60.13	55.5	44	20	12.16 ± 3.52	9.45 ± 1.52	Mixed	Mixed	GOLD I–IV
Aaron 2010	Canada	65.3–72.3	62.4–70.9	21	12	7.39 ± 6.32	2.3 ± 0.7	>10 pack-year	Nonsmokers	GOLD II–IV
Oncel 2010	Turkey	62.8	61.8	40	33	3.44 ± 2.68	3.04 ± 2.92	Mixed	Mixed	GOLD II–III
Ying 2008	China	69.79	67.70	38	24	3.43 ± 2.18	1.78 ± 1.08	Mixed	Mixed	No stage information
Gagnon 2014	Canada	65	62	37	19	5.04 ± 2.88	3.37 ± 2.19	≥15 pack-year	≥15 pack-year	GOLD I
Uzum 2013	Turkey	66.08	50.2	50	17	3.024 ± 3.17	1.2 ± 1.8	Male smokers	Nonsmokers	GOLD I–IV
Hageman 2003	Netherlands	62.6	62.1	37	21	217 ± 194.65	78 ± 45.83	Current smokers	Current smokers	No stage information
Ju 2012	Chinese	65.17	63.98	70	60	1.41 ± 0.37	1.34 ± 0.70	Ex-smokers	Smokers	GOLD II–IV
Walter 2008	UK	63.7	60.0	309	2244	4.9 ± 4.8	3.7 ± 5.0	Smokers	Smokers	No stage information
Higashimoto 2008	Japan	74.9	64.5	111	75	5.02 ± 5.58	2.65 ± 4.76	Mixed	Mixed	No stage information
Karadag 2008b	Turkey	65.6	63.2	35	30	67.51 ± 114.57	59.15 ± 100.38	Mixed	Nonsmokers	GOLD II–IV
Karadag 2008a	Turkey	65.54	64.10	83	30	68.86 ± 50.42	24.77 ± 47.23	Mixed	Mixed	GOLD II–IV
Hacker 2009	Austria	59.60	56.91	35	29	3.96 ± 5.944	3.48 ± 7.4	Mixed	Mixed	GOLD I–IV

**Notes.**

The word ‘Mixed’ means that the subjects (both cases and controls) were composed of smokers and nonsmokers.

GOLD, Global Initiative for Chronic Obstructive Lung Disease.

### Overall meta-analysis

**Figure 2:**
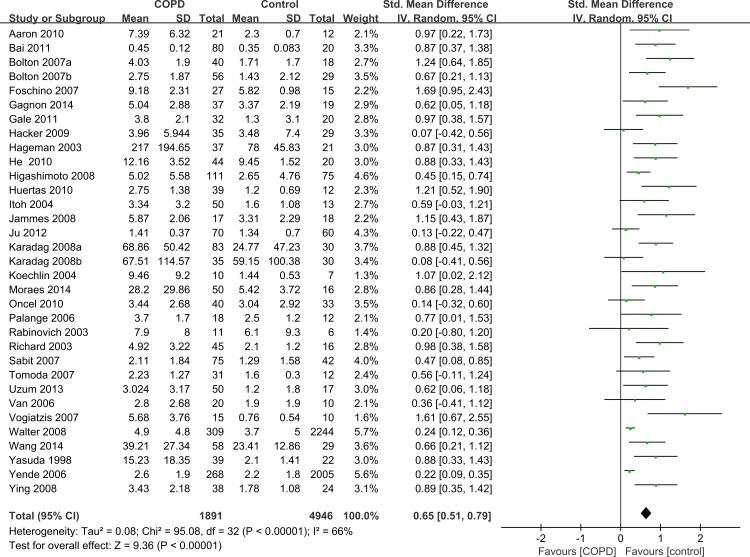
Forest plot for the association between serum IL-6 concentrations and COPD. Forrest plot of meta-analysis of 33 studies that investigated the association between serum IL-6 concentrations and COPD, using a random-effects model. COPD, chronic obstructive pulmonary disease; SD, standard difference; SMD, standard mean difference; CI, confidence interval.

Twelve articles contained subgroup analysis either in patients or healthy controls, and we combined these data by the combination formula wherever possible. The statistics which could not be combined were regarded as separate studies. A total of 33 studies which contained 1,891 stable COPD patients and 4,946 healthy controls were included. Since the methodological design in some studies was not the same, the SMD was selected for quantitative analysis. The studies showed that the serum levels of IL-6 were higher in stable COPD patients than healthy control subjects (SMD = 0.65, 95% CI [0.51–0.79], *P* < 0.00001; *I*^2^ = 66%, *P* < 0.00001; Fig. 2) (Aaron et al., 2010; Bai et al., 2011; Bolton et al., 2007a; Bolton et al., 2007b; de Moraes et al., 2014; Debigare et al., 2003; Foschino Barbaro et al., 2007; Gagnon et al., 2014; Gale et al., 2011; Hacker et al., 2009; Hageman et al., 2003; He et al., 2010; Higashimoto et al., 2008; Huertas et al., 2010; Itoh et al., 2004; Jammes et al., 2008; Ju & Chen, 2012; Karadag et al., 2008a; Karadag et al., 2008b; Koechlin et al., 2004; Oncel et al., 2010; Palange et al., 2006; Rabinovich et al., 2003; Sabit et al., 2007; Tomoda et al., 2007; Uzum et al., 2014; Van Helvoort et al., 2006; Vogiatzis et al., 2007; Walter et al., 2008; Wang et al., 2014; Yasuda et al., 1998; Yende et al., 2006; Ying et al., 2008).

### Subgroup analysis by comorbidities

**Figure 3:**
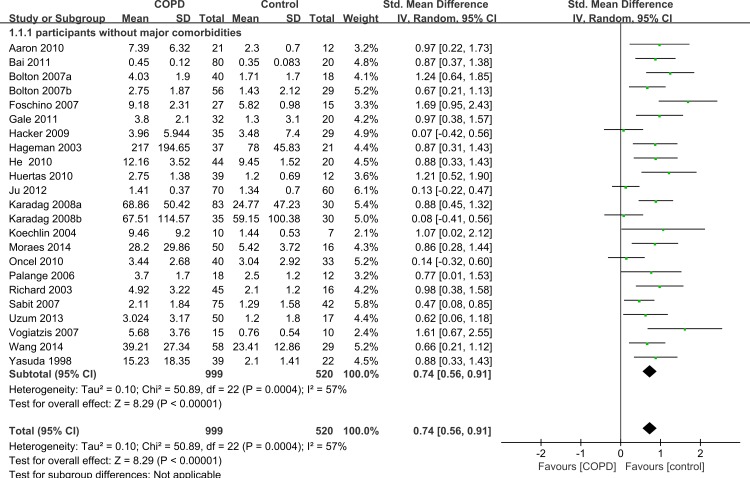
Forest plot for subgroup analysis by comorbidities. Forrest plot of subgroup studies in the meta-analysis that investigated the association between serum IL-6 concentrations and COPD patients without major comorbid conditions, using a random-effects model. COPD, chronic obstructive pulmonary disease; SD, standard difference; SMD, standard mean difference; CI, confidence interval.

Among the 33 selected studies, 26 mentioned comorbidities in their inclusion or exclusion criteria, and seven studies had no information about comorbidities (Itoh et al., 2004; Jammes et al., 2008; Rabinovich et al., 2003; Tomoda et al., 2007; Van Helvoort et al., 2006; Walter et al., 2008; Ying et al., 2008). A total of 23 of the 26 studies excluded participants with some major comorbidities such as liver disease, renal insufficiency, collagen disease, sarcoidosis, ischemic heart disease, congestive cardiac failure, cor pulmonale, severe coronary artery disease, malignancy, systemic autoimmune or connective tissue disorders, or any other inflammatory or metabolic condition. We conducted another subgroup analysis only including those 23 studies without major comorbidities and compared IL-6 concentrations between cases and controls. The results also showed that the serum levels of IL-6 were higher in stable COPD patients than healthy controls (SMD = 0.74, 95% CI [0.56–0.91], *P* < 0.00001; *I*^2^ = 57%, *P* = 0.0004; Fig. 3) (Aaron et al., 2010; Bai et al., 2011; Bolton et al., 2007a; Bolton et al., 2007b; de Moraes et al., 2014; Debigare et al., 2003; Foschino Barbaro et al., 2007; Gale et al., 2011; Hacker et al., 2009; Hageman et al., 2003; He et al., 2010; Huertas et al., 2010; Ju & Chen, 2012; Karadag et al., 2008a; Karadag et al., 2008b; Koechlin et al., 2004; Oncel et al., 2010; Palange et al., 2006; Sabit et al., 2007; Uzum et al., 2014; Vogiatzis et al., 2007; Wang et al., 2014; Yasuda et al., 1998).

### Subgroup analysis by disease severity

**Figure 4:**
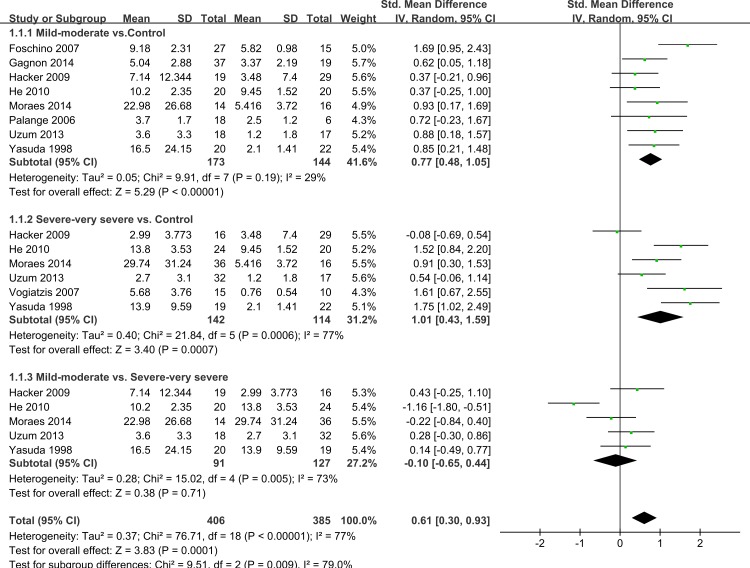
Forest plot for subgroup analysis by disease severity. Forrest plot of subgroup studies in the meta-analysis that investigated the association between serum IL-6 concentrations and pulmonary functions of COPD, using a random-effects model. COPD, chronic obstructive pulmonary disease; SD, standard difference; SMD, standard mean difference; CI, confidence interval.

Thirteen studies had subgroups according to smoking status, body mass index (BMI) and COPD severity. We only evaluated IL-6 levels by severity of COPD. COPD was defined as post-bronchodilator forced expiratory volume in one second (FEV_1_)/(forced vital capacity) (FVC) <70%. Depending on whether FEV_1_ was <50% predicted (GOLD stage 3–4) or >50% (GOLD stage 1–2) we called it severe–very severe COPD and mild-moderate COPD (GOLD stage 1–2) respectively. Nine studies showed data including mild-moderate and/or severe-very severe COPD (de Moraes et al., 2014; Foschino Barbaro et al., 2007; Gagnon et al., 2014; Hacker et al., 2009; He et al., 2010; Palange et al., 2006; Uzum et al., 2014; Vogiatzis et al., 2007; Yasuda et al., 1998) and five of them compared mild-moderate with severe-very severe COPD (de Moraes et al., 2014; Hacker et al., 2009; He et al., 2010; Uzum et al., 2014; Yasuda et al., 1998). We compared mild-moderate COPD and severe-very severe COPD with healthy controls, respectively. When compared mild-moderate COPD with healthy controls, we chose data from studies that included COPD patients of GOLD stage 1, GOLD stage 2 or GOLD stage 1 and 2, similarly with the severe-very severe group. It was found that COPD patients with an FEV_1_ either <50% predicted or >50% predicted had increased IL-6 concentrations compared with healthy control subjects (SMD = 0.77, 95% CI [0.48–1.05], *P* < 0.00001; *I*^2^ = 29%, *P* = 0.19; SMD = 1.01, 95% CI [0.43–1.59], *P* = 0.0006; *I*^2^ = 77%, *P* = 0.0007, respectively; Fig. 4). Another subgroup analysis compared the difference between mild-moderate and severe-very severe COPD patients, but no statistically significant result was seen (SMD = − 0.1, 95% CI [−0.65–0.44], *P* = 0.71; *I*^2^ = 73%, *P* = 0.005; Fig. 4).

### Sensitivity analysis

We performed a sensitivity analysis for statistically significant result. Among the overall studies, the observed significant result was not materially altered after sequentially excluding each study. In the last subgroup comparing mild-moderate and severe-very severe COPD patients, at the time of data extraction, the result was influenced by the study of He et al. (2010), so it was considered to be a result of heterogeneity. After excluding this study, the heterogeneity significantly decreased (SMD = 0.15, 95% CI [−0.16–0.46], *P* = 0.35; *I*^2^ = 0%, *P* = 0.53).

### Heterogeneity and publication bias

**Figure 5:**
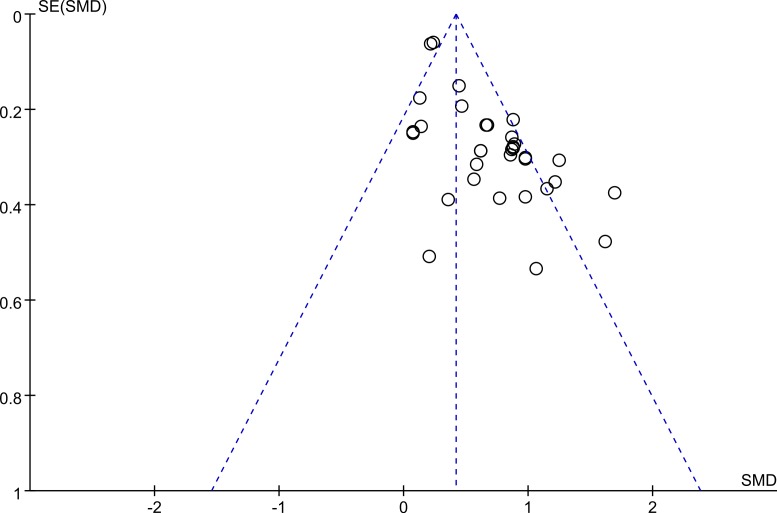
Funnel plot. Funnel plot for evaluation of publication bias in the included studies on the association between serum IL-6 concentrations and COPD. COPD, chronic obstructive pulmonary disease; SMD, standard mean difference.

Egger’s test showed a publication bias in the overall meta-analysis (*P* < 0.00001), and the shape of the funnel plot was asymmetrical (Fig. 5), and so as the subgroup analysis of participants without major comorbidities. This might be explained by the presence of a language bias, inflated estimates by a flawed methodological design in smaller studies, lack of small trials with opposite results as well as the ethnicity difference since two of the studies, Walter et al. (2008) (from the UK) and Yende et al. (2006) (from the USA) possess largest cohort size. However, there was no significant evidence of publication bias among mild-moderate COPD versus controls (*P* = 0.231), severe-very severe COPD versus healthy controls (*P* = 0.137), and mild-moderate COPD versus severe-very severe COPD (*P* = 0.769).

## Discussion

COPD is characterized by an intense inflammatory process in the airways, parenchyma, and pulmonary vasculature. Chronic inflammation leads to fixed narrowing of small airways and alveolar wall destruction (emphysema) (Barnes, Shapiro & Pauwels, 2003). Further studies have found that the inflammatory response not only existed in the airway and lung itself (Hogg et al., 2004), but also in the systemic circulatory system (Gan et al., 2004). Moreover, the systemic inflammatory response in acute exacerbation phase is significantly enhanced (Hurst et al., 2006; Stolz et al., 2007) and the production of TNF-*α*, interleukin-type (IL-6, IL-8), CRP and other inflammatory cytokines increases in the circulatory system (Gan et al., 2004; Rosenberg & Kalhan, 2012).

IL-6 is a crucial cytokine, which can produce a variety of acute phase proteins when it acts on liver cells (Heinrich, Castell & Andus, 1990). Some observational studies indicated the IL-6 levels were significantly elevated in the peripheral blood of patients with COPD (Garcia-Rio et al., 2010; Tanni et al., 2010; Yasuda et al., 1998), and it was associated with FEV_1_ (Garcia-Rio et al., 2010). However, other studies (Bai et al., 2011; Huertas et al., 2010) showed no statistical significance. Therefore, we performed this comprehensive meta-analysis that included the latest data, to examine the associations between IL-6 levels and stable COPD. Our findings demonstrated that concentrations of serum IL-6 were higher in stable COPD than healthy controls, and the serum IL-6 concentrations might have no association with pulmonary function impairment.

The results of the pooled analysis were consistent with the findings of some previous studies (Bolton et al., 2007a; Bolton et al., 2007b; de Moraes et al., 2014; Foschino Barbaro et al., 2007; Gale et al., 2011). It demonstrated that IL-6 blood levels were significantly elevated in patients with COPD compared to those in healthy subjects, which suggests that systemic inflammatory activity exists in stable COPD patients. A study by Oncel et al. (2010) showed no significant difference in the values of IL-6 between patients and controls which differed from our result. Itoh et al. (2004) reported that although the plasma IL-6 levels were higher in both normal weight patients and control subjects, it did not significantly differ between them. Likewise, Van Helvoort et al. (2006) also reported similar results that inflammatory values did not reach significance between muscle-wasted and non–muscle-wasted patients. These findings reflect that increased IL-6 may induce a catabolic response in tissues and can trigger muscle proteolysis, leading to an increase in protein degradation. Obvious heterogeneity was observed in the comparison of our study which might be explained by methodological differences among those primary studies, especially gender differences and different disease severity in the included population.

Despite the concept that pulmonary inflammation might be more likely to become persistent and may result in substantial extra-pulmonary manifestations leading to more pronounced increases in serum IL-6 concentrations in severe COPD patients, we found that patients with even mild-moderate airway obstruction had increased IL-6 levels compared with healthy controls, and no difference was seen in plasma IL-6 levels between the mild-moderate group and severe–very severe group. This result was not consistent with the cross-sectional study of Sabit et al. (2007) who found patients with an FEV_1_ > 50% predicted had lower IL-6 levels than patients with an FEV_1_ < 50% predicted (*p* < 0.05). Reasons for this difference might be the small number of Sabit’s study (75 COPD patients and 42 controls) and the difference in methods used for detecting serum samples. Our study contained 1891 COPD patients and 4,946 controls and the results may thus be more reliable. Similar to our finding, Yasuda et al. (1998) reported that plasma IL-6 concentrations increased in severe and mild/moderate COPD compared to the healthy controls, but no significant difference was found between severe and mild/moderate COPD. It is a meaningful result which highlights the early systemic inflammation in stable COPD patients.

The serum IL-6 concentration of COPD is usually not monitored unless an exacerbation is suspected or occurs concurrently. The present meta-analysis gave us the impression that serum levels of IL-6 increased even in mild COPD and might be a better marker of the early inflammation and associated comorbidities. IL-6 participates directly in inflammation, which may be regarded as a marker of low-grade systemic inflammation and an additional parameter for risk assessment together with smoking, number of exacerbations, hospitalization rate and mortality rate. More evidence of early interventions might be obtained by synthesizing all these parameters, thus decreasing the risk of possible complications.

The limitations of this meta-analysis should be emphasized. Firstly, the publication bias was detected in our study, but it was impossible for us to adjust the impact of these confounding factors such as smoking status, age, gender, BMI, COPD phenotype, frequency of exacerbations, time course of pulmonary function loss, and treatment information, which could influence the concentrations of IL-6, and the control subjects were not matched for these confounders either. It is important to minimize selection bias in future studies. Secondly, some studies had subgroup analysis and we used formulas to calculate these data without considering the confounding factors, which may have lead to lower statistical power. Thirdly, some studies were of a small scale, which may affect the power to explore the real association. Lastly, nearly all the included studies were conducted in different institutions, thus different methods or kits might have been used for measuring the serum IL-6 levels, and the detection limit varied, which could inconspicuously influence the data.

## Conclusions

In conclusion, this is the most comprehensive meta-analysis to date to show that serum IL-6 concentrations are higher in stable COPD patients than healthy controls, but no evidence shows positive or negative association between IL-6 concentration and the severity of pulmonary function impairment. The correlation between IL-6 levels and pulmonary function tended to be weak in different severities of stable COPD patients. These findings may partly explain the high prevalence of systemic complications such as cachexia, anorexia and atherosclerosis of COPD. These may also indicate that systemic inflammation occurs in an early stage of stable COPD, so it is valuable to monitor serum IL-6 concentrations for early detection of pulmonary function impairment. In this regard, more studies with larger sample sizes and those including different stages to evaluate the serum IL-6 levels and disease severity are needed to better identify the role of serum IL-6. Whether early interventions would decrease the serum IL-6 levels and modify the risk of complications in COPD should also be determined in future studies.

## Supplemental Information

10.7717/peerj.1199/supp-1 Supplemental Information 1 PRISMA 2009 checklist

## Additional Information and Declarations

### Data Availability

The following information was supplied regarding the deposition of related data: All relevant raw data appears in Table 1.
